# Valorization of *Pistacia lentiscus* L. Hydrodistillation By-Products: Phytochemical Profile and Multitarget Anti-Aging Activity of an Aqueous Extract

**DOI:** 10.3390/plants15071013

**Published:** 2026-03-26

**Authors:** Antonella Fais, Francesca Pintus, Benedetta Era, Sonia Floris, Giulia Urru, Enrico Sanjust, Emma Cocco, Andrea Maxia, Valentina Masala, Carlo Ignazio Giovanni Tuberoso

**Affiliations:** 1 Department of Life and Environmental Sciences, University of Cagliari, Cittadella Universitaria, SS 554, 09042 Monserrato, Italy; fais@unica.it (A.F.); fpintus@unica.it (F.P.); era@unica.it (B.E.); s.floris@unica.it (S.F.); valentina.masala2@unica.it (V.M.); tuberoso@unica.it (C.I.G.T.); 2 Department of Biomedical Sciences, University of Cagliari, Cittadella Universitaria, SS 554, 09042 Monserrato, Italy; 3 Laboratory of Economic and Pharmaceutical Botany, Department of Life and Environmental Sciences, University of Cagliari, V.le S. Ignazio da Laconi 13, 09123 Cagliari, Italy; andrea.maxia@unica.it

**Keywords:** *Pistacia lentiscus*, skin aging, tyrosinase, elastase, hyaluronidase, hydrodistillation by-products, enzyme inhibition, LC-ESI-QToF-MS/MS

## Abstract

*Pistacia lentiscus* L. is widely reported in the ethnobotanical literature for its use in treating various pathologies, particularly skin disorders such as burns, inflammation, and wounds. These traditional applications suggest broader potential anti-aging activity and support the exploration of simple and sustainable extraction strategies. In this study, an aqueous extract obtained from leaf hydrodistillation residues, representing a by-product of essential oil production, was chemically characterized by LC-ESI-QToF-MS/MS and HPLC-PDA. The analysis identified 32 compounds, including 8 gallic acid derivatives, accounting for 70.7% of the total extract, and 24 flavonol glycosides. High total phenolic and flavonoid contents were associated with strong antioxidant activity, confirmed by the ABTS assay and by a dose-dependent intracellular ROS reduction in keratinocytes. The extract exhibited significant inhibitory activities against key skin-aging-related enzymes, with IC_50_ of 33.8 µg/mL and 17.4 µg/mL, respectively for tyrosinase and elastase. Notably, strong anti-hyaluronidase activity, IC_50_ 4.3 µg/mL, is reported here for the first time, while no collagenase inhibition was observed. The cytotoxicity assay demonstrated a favorable safety profile at biologically active concentrations. Overall, these results highlight the bioactivity of the *P. lentiscus* phenolic phytocomplex and support the valorization of hydrodistillation aqueous residues as a sustainable source of multifunctional bioactive compounds for dermocosmetic applications.

## 1. Introduction

Skin aging is a multifactorial biological process marked by a progressive decline in cellular and tissue homeostasis, influenced by both intrinsic mechanisms and extrinsic stressors such as ultraviolet radiation, pollution, smoking, and diet [1,2]. A key molecular feature of both intrinsic and extrinsic aging is the dysregulation of enzymes involved in extracellular matrix (ECM) remodeling and melanogenesis [3]. In particular, tyrosinase, elastase, collagenase and hyaluronidase are critically implicated in age-related alterations of skin structure and function, and their activity is strongly modulated by oxidative and inflammatory stimuli [4]. Tyrosinase catalyzes the rate-limiting steps of melanogenesis, including the hydroxylation of L-tyrosine to L-DOPA and its subsequent oxidation to dopaquinone; excessive tyrosinase activity is closely associated with hyperpigmentation disorders [5]. Elastase and collagenase contribute to the degradation of elastin and collagen fibers, respectively, leading to the loss of skin elasticity, firmness, and tensile strength [6,7]. Hyaluronidase, by hydrolyzing hyaluronic acid, compromises skin hydration, viscoelasticity, and turgor, further exacerbating the aging phenotype [8]. Collectively, the altered activity of these enzymes affects multiple skin layers, from the epidermis to deeper dermal compartments, and is associated with various dermatological conditions and pigmentation disorders, including psoriasis, dermatitis, melasma, and senile lentigo [8].

These considerations underscore the rationale for multitarget therapeutic strategies aimed at modulating enzyme activity in anti-aging interventions. In this context, a wide range of plant-derived bioactive molecules, particularly phenolic compounds and other secondary metabolites, have attracted increasing scientific interest due to their ability to counteract aging-related mechanisms through multiple and complementary modes of action [9,10,11].

In this context, *Pistacia lentiscus* L. [12], an evergreen shrub characteristic of the Mediterranean maquis, has a long-standing history of medicinal use, documented by ethnobotanical knowledge, and represents a particularly valuable source of bioactive compounds. It is widely reported for its extensive traditional applications in the treatment of various disorders, including skin infections, burns, and wounds [13,14]. Boualsa et al. [15] highlighted its preferential use in the management of skin-related illnesses, commonly applied as macerated preparations. In Sardinia, the external use of *P. lentiscus* has also been documented in ethnoveterinary practices, particularly for wound healing and the treatment of scabies [16].

Phytochemical and pharmacological investigations have consistently demonstrated that essential oils and extracts derived from *P. lentiscus* exhibit a wide range of biological activities, including antioxidant, anti-inflammatory, antimicrobial, antidiabetic and anticancer effects [17,18,19,20,21]. Detailed chemical profiling has revealed a high abundance of polyphenols, flavonoids, and other bioactive secondary metabolites, which are widely regarded as the principal contributors to these pharmacological properties [22].

In particular, residues obtained from essential oil production, such as those remaining after hydrodistillation, constitute an underexploited reservoir of polyphenols, flavonoids, and other secondary metabolites [23]. This consideration is especially pertinent in light of the typically low yields of essential oils. In the case of *P. lentiscus*, yields range from approximately 0.30% under optimal seasonal conditions to below 0.02%, indicating that the production of 100 mL of essential oil may result in the generation of approximately 500 kg of residual plant material [24]. The large volume of such by-products underscores their considerable potential for valorization as a sustainable and readily available source of bioactive compounds.

With regard to the enzymes involved in skin aging, to the best of our knowledge, no investigations have reported the anticollagenase or antihyaluronidase activities of *P. lentiscus* extracts. Conversely, Chiocchio et al. [25] and Elloumi et al. [23] provided preliminary evidence regarding its anti-tyrosinase and anti-elastase activities. Chiocchio et al. [25] demonstrated that methanolic leaf extracts exerted marked inhibitory effects, achieving inhibition rates of 78% against tyrosinase and 74% against elastase, with corresponding IC_50_ values of approximately 40 µg/mL and 7 µg/mL, respectively. Elloumi et al. [23] reported that the ethyl acetate fraction exhibited low tyrosinase inhibitory activity while maintaining a pronounced inhibitory effect against elastase, with IC_50_ values of 123 µg/mL and 19 µg/mL, respectively. However, the majority of these investigations relied on extraction procedures employing organic solvents, such as methanol or ethyl acetate, which are associated with environmental concerns and potential toxicological risks. The use of such solvents limits the direct applicability of the resulting extracts in skin-related formulations and raises safety issues, particularly in the context of invitro cytotoxicity and cell viability assays. Moreover, several studies on *P. lentiscus* demonstrated that water represents a more efficient and sustainable extraction solvent, providing higher extraction yields, enhancing the recovery of flavonoids and polyphenols, and exhibiting superior biological activity [26,27]. This effect may also be related to the extraction capability of water, resulting in a more hydrophilic chemical composition. Furthermore, aqueous extraction methods are more consistent with the traditional uses of *P. lentiscus* documented in the ethnobotanical literature [15,16].

Therefore, the present study aims to investigate the multitarget anti-aging potential of *P. lentiscus* by-product, specifically the water residue remaining after leaf hydrodistillation (WEDR). This by-product, typically considered a waste stream of the essential oil extraction process, represents a valuable secondary output that can be recovered and valorized. Here, it is obtained through a simple, sustainable, and non-toxic extraction methodology based exclusively on water as the extraction solvent. WEDR was then evaluated for its antioxidant capacity, total phenolic and flavonoid contents, and inhibitory activity against four key enzymes involved in skin aging, namely tyrosinase, elastase, collagenase and hyaluronidase, thus enabling direct comparison with previously reported studies. In addition, WEDR’s chemical profile was characterized by LC-MS analysis, and its cytotoxic potential was assessed to further support its safety profile. Through this approach, the study aims to valorize a *P. lentiscus* by-product as a source of bioactive compounds with anti-aging activity, and to provide novel insights into sustainable and effective strategies for skin-related applications.

## 2. Results

### 2.1. Quali-Quantitative Determination of Phenolic Compounds in P. lentiscus Leaves Water Extract–Distillation Residues

*P. lentiscus* WEDR was obtained as a homogeneous liquid extract, with a characteristic green-brown color. Following filtration to remove residual plant material, *P. lentiscus* WEDR was qualitatively analyzed by (HR) LC-ESI-QToF-MS/MS in negative ion mode, and targeted phenolic compounds were quantified by LC-PDA analysis.

**Figure 1:**
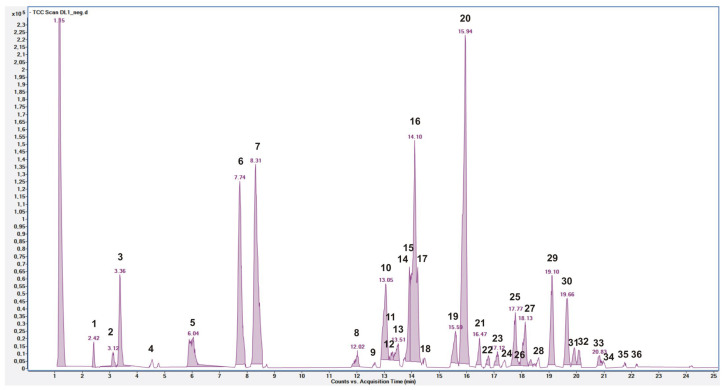
(HR) LC-ESI-QToF-MS Total Compound Chromatogram of *P. lentiscus* WEDR acquired in negative ion mode. Chromatographic conditions are described in the methods section. Above each peak, the corresponding compound number is indicated in black, as defined in Table 1.

The negative LC-MS profile highlighted the presence of a large group of compounds (Figure 1), and individual components were identified by comparison of their *m*/*z* values in the total compound chromatogram (TCC) profile with those of the selected compounds described in the literature, and by comparing experimental MS/MS spectra with fragmentation patterns reported in the literature for the same analytes or with the fragmentation patterns and spectra reported in a public repository of mass spectral data [28,29,30].

Table 1 reports the compounds identified by High-Resolution Mass Spectrometric Data, listed according to their retention times, the chemical formula derived by accurate mass measurement (experimental results), MS/MS results, mass error (Δ ppm), the references used for identification and the identification confidence levels [31]. Thirty-two compounds were tentatively identified as galloyl and flavonoid derivatives, and the other four remained unknown. Table 2 reports the ʎ_max_, evaluated in the range 200–600 nm, used to support the identification of targeted phenolic compounds detected in the *P. lentiscus* WEDR and their concentration (mg/L).

**Table 1:** Compounds identification by (HR) LC-ESI-QToF-MS/MS in *P. lentiscus* WEDR.

Compound n°	Rt min	Identity	[M-H]^−^ *m*/*z*	Molecular Formula	Δ ppm	MS/MS * m/*z*	References	Level #
**1**	2.42	Galloyl quinic acid isomer I	343.0670	C_14_H_16_O_10_	−0.46	191.0557(100)	[32,33]	2
**2**	3.12	Gallocatechin-(epi)gallocatechin	609.1247	C_30_H_26_O_14_	−0.68	423.0731(77)/334.0239(80)/177.0181(81)	[32]	2
**3**	3.36	Galloyl quinic acid isomer II	343.0670	C_14_H_16_O_10_	−0.42	191.0557(100)/169.0155(11)	[32,33]	2
**4**	4.53	Gallocatechin	305.0664	C_15_H_14_O_7_	−0.67	167.0352(59)/137.0253(100)/125.0235(21)	[32,33]	1
**5**	6.04	Digalloyl quinic acid isomer I	495.0779	C_21_H_20_O_14_	−0.25	343.0651(25)/191.0546(35)/169.0130(100)	[32,33]	2
**6**	7.74	Digalloyl quinic acid isomer II	495.0782	C_21_H_20_O_14_	0.33	343.0660(18)/191.0550(68)/169.0142(100)	[32,33]	2
**7**	8.31	Digalloyl quinic acid isomer III	495.0779	C_21_H_20_O_14_	0.24	343.0671(40)/191.0561(9)/169.014(100)	[32]	2
**8**	12.02	Unknown	381.1766	C_16_H_30_O_10_	0.94			4
**9**	12.64	Epigallocatechin gallate	457.0774	C_22_H_18_O_11_	−0.83	318.0776(88)/167.0238(21)/137.0266(100)	[32,33]	1
**10**	13.05	Unknown	332.1835	C_16_H_28_O_7_	0.02			4
**11**	13.13	Myricetin-galloylglucoside isomer I	631.0936	C_28_H_24_O_17_	−0.74	479.0809(93)/316.0195(100)/271.0205(29)	[34,35]	2
**12**	13.31	Myricetin-galloylglucoside isomer II	631.0939	C_28_H_24_O_17_	−0.26	479.0835(48)/316.0220(80)/271.0205(29)	[34,35]	2
**13**	13.50	Unknown	396.1991	C_17_H_32_O_10_	−1.00			4
**14**	13.91	Myricetin hexoside isomer I	479.0830	C_21_H_20_O_13_	−0.13	317.0260(24)/316.0231(100)	[32]	2
**15**	14.03	Myricetin glucuronide	493.0624	C_21_H_18_O_14_	0.42	317.0298(100)/123.0091(76)	[33]	2
**16**	14.10	Myricetin rutinoside	625.1414	C_27_H_30_O_17_	0.55	317.0296(39)/316.0220(100)	[33]	2
**17**	14.21	Myricetin-hexoside isomer II	479.0832	C_21_H_20_O_13_	0.16	316.0231(100)/271.0240(34)	[32]	2
**18**	14.47	Quercetin hexoside	463.0882	C_21_H_20_O_12_	−0.11	301.0311(100)/300.0254(85)	[32]	2
**19**	15.59	Myricetin pentoside	449.0724	C_20_H_18_O_12_	0.07	317.0269(11)/316.0216(100)	[32]	2
**20**	15.94	Myricetin rhamnoside	463.0886	C_21_H_20_O_12_	0.81	317.0277(34)/316.0224(100)	[32]	2
**21**	16.46	Quercetin dihexoside (rut)	609.1459	C_27_H_30_O_16_	−0.78	300.0773(66)/271.0253(48)/179.1290(20)	[28]	2
**22**	16.78	Quercetin hexoside	463.0882	C_21_H_20_O_12_	−0.11	317.0293(35)//300.0254(100)	[32]	2
**23**	17.12	Kaempferol or luteolin hexoside	447.0935	C_21_H_20_O_11_	0.26	285.0406(100)	[32]	2
**24**	17.36	Quercetin galloyl hexoside isomer I	615.0994	C_28_H_24_O_16_	0.41	301.054(100)/273.0418(72)/179.0001(46)	[36]	2
**25**	17.77	Quercetin galloyl hexoside isomer II	615.0994	C_28_H_24_O_16_	−0.03	301.0339(100)/300.0295(24)	[36]	2
**26**	18.04	Quercetin pentoside	433.0782	C_20_H_18_O_11_	0.89	300.0267(100)/271.0247(57)	[34]	2
**27**	18.13	Quercetin-galloyl rutinoside	761.1573	C_34_H_34_O_20_	0.19	497.1634(10)/301.0372(100)/178.9983(22)	[28]	2
**28**	18.60	Kaempferol or luteolin rutinoside	593.1508	C_27_H_30_O_15_	−0.62	285.0388(100)/284.0340(79)/255.0249(73)	[36]	2
**29**	19.10	Quercetin ramnoside (quercitrin)	447.0935	C_21_H_20_O_11_	0.36	301.0337(65)/300.0276(100)/271.0249(22)	[33]	2
**30**	19.66	Unknown	407.0845	C_19_H_32_O_7_	−0.99	217.0416(96)		4
**31**	19.92	Quercetin galloyl-pentoside	585.0886	C_27_H_22_O_15_	−0.03	301.0376(63)/172.9341(75)/151.0004(100)	[32,33]	2
**32**	20.10	Kaempferol galloyl-hexoside	599.1046	C_28_H_24_O_15_	0.17	285.0402(100)	[37]	2
**33**	20.83	Kaempferol or luteolin hexoside	447.0936	C_21_H_20_O_11_	0.68	285.0389(100)	[32]	2
**34**	20.97	Myricetin galloyl-deoxyhexoside	615.0990	C_28_H_24_O_16_	0.41	317.0301(100)/191.0439(60)/151.0038(65)	[32]	2
**35**	21.76	Kaempferol or luteolin deoxyhexoside	431.0976	C_21_H_20_O_10_	−1.02	285.0422(80)/284.0320(53)/255.0278(100)	[32]	2
**36**	22.19	Kaempferol galloyl-pentoside	569.0936	C_27_H_22_O_14_	−0.88	285.0397(71)/257, 229, 151	[37]	2

* in brackets the relative intensity; # according to Blaženović et al. [31].

**Table 2:** Concentration of targeted phenolic compounds detected in *P. lentiscus* WEDR (mg/L, mean ± SD *n* = 3).

Compound	Peak No. ^§^	ʎ_max_	*P. lentiscus* WEDR (mg/L)	
			Mean	±SD
**Total galloyl derivatives**			47.29	3.11
Galloyl quinic acid isomer I ^a^	1	216/274	14.79	1.27
Gallocatechin-(epi)gallocatechin ^a^	2	208/276	0.18	0.02
Galloyl quinic acid isomer II ^a^	3	216/274	7.64	0.14
Gallocatechin	4	206/274	0.58	0.04
Digalloyl quinic acid isomer I ^a^	5	216/276	1.99	0.09
Digalloyl quinic acid isomer II ^a^	6	216/276	12.09	0.88
Digalloyl quinic acid isomer III ^a^	7	216/277	8.39	0.31
Epigallocatechin gallate	9	208/276	1.63	0.10
**Total Flavonols**			19.55	0.89
Myricetin-galloylglucoside I ^b^	11	212/270/356	0.07	0.00
Myricetin-galloylglucoside II ^b^	12	212/270/356	0.07	0.00
Myricetin-hexoside ^b^	14	208/260/356	3.22	0.08
Myricetin-rutinoside	16	208/260/356	3.85	0.13
Myricetin pentoside ^b^	19	208/260/354	0.08	0.00
Myricetin ramnoside	20	208/261/350	6.82	0.27
Quercetin dihexoside ^c^	21	204/257/354	0.64	0.05
Quercetin hexoside ^c^	22	204/256/353	0.68	0.03
Quercetin galloyl hexoside isomer I ^c^	24	208/255/352	0.08	0.01
Quercetin galloyl hexoside isomer II ^c^	25	209/254/353	0.25	0.02
Quercetin pentoside ^c^	26	204/255/354	0.30	0.02
Quercetin-galloyl rutinoside ^c^	27	209/254/354	0.28	0.04
Kaempferol or luteolin rutinoside	28	201/264/348	0.28	0.02
Quercetin rhamnoside (quercitrin)	29	204/256/349	1.37	0.10
Quercetin galloyl-pentoside ^c^	31	209/254/355	0.55	0.03
Kaempferol galloyl-hexoside ^d^	32	214/270/349	0.39	0.03
Kaempferol or luteolin hexoside ^d^	33	202/265/348	0.24	0.02
Myricetin galloyl-deoxyhexoside ^b^	34	210/268/350	0.09	0.02
Kaempferol or luteolin deoxyhexoside ^d^	35	203/264/346	0.14	0.01
Kaempferol galloyl-pentoside ^d^	36	210/264/350	0.15	0.01
**Total polyphenols**			66.84	4.03

^a^ expressed as gallic acid equivalents; ^b^ expressed as myricetin-3-*O*-ramnoside equivalents; ^c^ expressed as quercetin equivalents; ^d^ expressed as kaempferol; ^§^ peak number as reported in Table 1.

Eight peaks were identified as galloyl derivatives, namely galloyl quinic acids and catechins [32,33]. Quinic acid derivatives are characterized by a deprotonated molecule at *m*/*z* 191, and the generation of product ions at *m*/*z* 169 corresponds to the neutral loss of a gallic acid moiety. All these compounds accounted for 70.7% (47.29 ± 3.11 mg/L) of total phenolic compounds, with galloyl quinic acid isomer I and digalloyl quinic acid isomer II the most concentrated compounds (14.79 ± 1.27 and 12.09 ± 0.88 mg/L, respectively).

Twenty-four peaks were attributed to flavonol derivatives, among which 10 originated from quercetin (18, 21, 22, 24–27, 29, 31 and 32), 9 from myricetin (11, 12, 14–17, 19, 20 and 34), and kaempferol/luteolin (23, 28, 33, 35 and 36). The type of flavonol derivatives included glycosides and galloyl-glycosides of mono- and di-glycosides of both hexoses and pentoses.

From a quantitative point of view, the total flavonol amount was 19.55 ± 0.89 mg/L (Table 2), with myricetin derivatives accounting for 73%, followed by quercetin and kaempferol derivatives (21 and 6%, respectively). Myricetin-rhamnoside was the most abundant flavonol (6.82 ± 0.27 mg/L), followed by myricetin-rutinoside and myricetin-hexoside.

### 2.2. Phenolic and Flavonoid Contents and Antioxidant Activity

*P. lentiscus* WEDR was analyzed for its total phenolic and flavonoid contents. As reported in Table 3, the extract shows a high concentration of both total phenols and total flavonoids. The concentrations are higher than those previously reported for *P. lentiscus* extracts obtained using methanol as the extracting solvent [23].

The antioxidant potency, as measured using the ABTS assay, was higher than that previously obtained in the aqueous extract of *P. lentiscus* (IC_50_ = 124 μg/mL) [27]. However, it remained lower than that of the reference compound Trolox (Table 3).

### 2.3. Anti-Aging Activity

The anti-aging properties of *P. lentiscus* WEDR were evaluated using enzyme inhibition assays targeting enzymes responsible for degrading ECM macromolecules: elastase, hyaluronidase and collagenase. The aging process is also associated with changes in skin pigmentation (such as age spots or hyperpigmentation), so the extract’s inhibitory properties against tyrosinase were also evaluated (Table 4).

The WEDR shows good inhibitory activity against tyrosinase and elastase, although the IC_50_ values are higher than those of the respective standard inhibitors. The *P. lentiscus* WEDR exhibited higher tyrosinase inhibition activity compared to the ethyl acetate extract [23], which shows an IC_50_ of 123 µg/mL. An additional tyrosinase inhibition assay [40] was performed using a catechol/aromatic amine–based method instead of using a dopachrome-based assay. This method was employed to avoid potential interference due to phenolics’ interaction with dopaquinone, thus preventing dopachrome formation, potentially leading to false-positive results in dopachrome-based measurements. The results obtained with this complementary assay qualitatively confirmed the inhibitory activity of the *P. lentiscus* WEDR, by the formation of the characteristic purplish-blue adduct between 4-methyl-1,2-benzoquinone (arising from the enzyme action on methylcatechol) and the aromatic diamine. This demonstrated that the observed effect is attributable to true tyrosinase inhibition rather than to nonspecific antioxidant or quinone-scavenging mechanisms.

The IC_50_ value of the WEDR against hyaluronidase was significantly lower than that of oleanolic acid (*p* < 0.0001), showing an inhibitory activity 22.6-fold higher than that of oleanolic acid used as a standard inhibitor.

Given that the standard inhibitor is a single molecule, while the extract contains numerous compounds, the observed activity is particularly encouraging.

The extract shows only minimal inhibitory activity against collagenase (IC_50_ > 150 µg/mL).

### 2.4. Cell Viability

**Figure 2:**
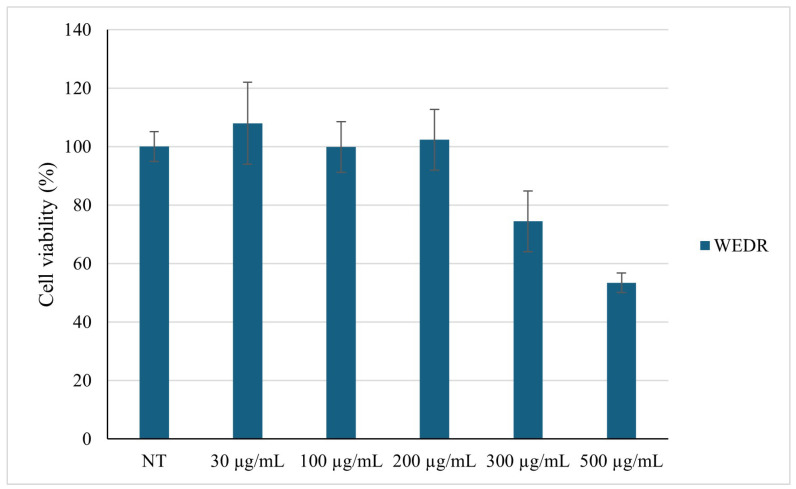
Effect of *P. lentiscus* WEDR on HaCat cell viability using the MTT assay. Cells were treated with different concentrations of the WEDR. Data are expressed as a percentage of the control. Data represent the mean (±standard deviation) of six independent experiments.

In order to determine the safety of WEDR, cells were treated with various concentrations of the sample for 24 h and were examined using the MTT test (Figure 2).

**Figure 3:**
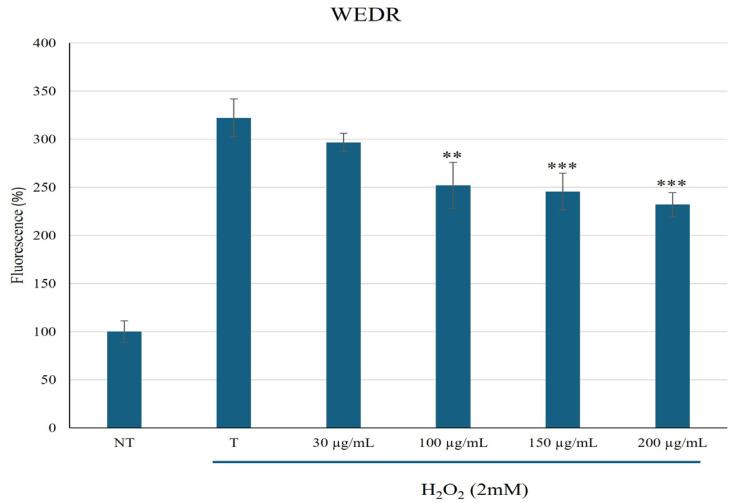
ROS levels (expressed as fluorescence) in HaCat cells pretreated with different concentrations of *P. lentiscus* WEDR and incubated with 2 mM of H_2_O_2_ up to 120 min. NT, non-treated cells; T, cells treated with H_2_O_2_ only. Data represent the mean (±standard deviation) of six independent experiments. Asterisks indicate values statistically different from cells treated with H_2_O_2_ only (T), based on one-way ANOVA followed by Tukey’s post hoc test. The following *p*-values were obtained for the WEDR concentrations: 100 μg/mL (** *p* < 0.0021), 150 μg/mL and 200 μg/mL (*** *p* < 0.001).

Since the viability was not affected until 200 µg/mL, we decided to conduct further cellular experiments using up to this extract concentration. We evaluated ROS levels in the cells before and after oxidative stress, and after treatment with the extract. As shown in Figure 3, incubation with H_2_O_2_ significantly increased ROS formation in HaCaT cells; however, treatment with the extract inhibited H_2_O_2_-induced ROS production in a dose-dependent manner. Thus, these results corroborate the antioxidant assays and suggest that the extract may also reduce ROS formation in cells.

## 3. Discussion

The present study demonstrates that aqueous residues obtained after hydrodistillation of *Pistacia lentiscus* L. leaves represent a valuable and underexploited source of bioactive compounds with multitarget anti-aging potential. The extraction strategy adopted herein, based exclusively on water and integrated into an essential oil production process, proved to be simple, effective and sustainable, enabling the recovery of a phenolic-rich extract without the use of organic solvents. This approach aligns with green chemistry principles and provides a concrete example of by-product valorization within a circular economy framework.

The chemical analysis revealed that the qualitative composition of the phenolic fraction recovered from post-hydrodistillation aqueous residues closely mirrors that reported in the literature for *P. lentiscus* leaf extracts obtained through maceration or organic solvent extraction [41]. In particular, galloyl derivatives and flavonol glycosides dominated the profile, confirming that thermal treatment and prolonged contact with water do not substantially alter the chemical identity of the major polyphenolic constituents. These findings are consistent with previous reports by Pacifico et al. [32] and Boucheffa et al. [33] and indicate that hydrodistillation does not induce significant degradation or transformation of the phenolic fraction.

Quantitatively, the WEDR exhibited notably high levels of total phenols and flavonoids, exceeding those previously reported for methanolic extracts of *P. lentiscus*. Galloyl derivatives accounted for approximately 70% of the total phenolic content, with galloyl- and digalloyl-quinic acids representing the most abundant compounds, while flavonols, particularly myricetin derivatives, constituted the remaining fraction. Specifically, galloyl quinic acid isomer I (14.79 mg/mL) and digalloyl quinic acid isomer II (12.09 mg/mL) were identified as the most dominant constituents, followed by digalloyl quinic acid isomer III (8.39 mg/mL) and galloyl quinic acid isomer IIa (7.64 mg/mL). Among flavonoids, myricetin rhamnoside was also found to be a relevant component, with a concentration of 6.82 mg/mL. Galloylated structures are known to enhance radical scavenging capacity and protein-binding affinity, which may be particularly relevant for enzyme inhibition mechanisms [42,43,44].

The total phenolic and flavonoid contents of the WEDR were higher than those previously reported for *P. lentiscus* extracts, regardless of the extraction solvent employed, including water, methanol, or ethyl acetate [23,27]. These elevated levels are reflected in the pronounced antioxidant activity observed in the present study, which exceeded values reported in the literature and was further corroborated by the effective reduction in intracellular ROS levels in keratinocytes subjected to oxidative stress. This finding confirms the well-established correlation between phenolic abundance and antioxidant efficacy and underscores the relevance of this extract in counteracting oxidative stress, a central driver of skin aging and the dysregulation of enzyme-mediated processes.

Beyond antioxidant effects, the WEDR exhibited significant inhibitory activity against key enzymes involved in skin aging, namely tyrosinase and elastase. The tyrosinase inhibitory activity observed in the present study (IC_50_ = 33.78 µg/mL) is comparable to that reported by Chiocchio et al. [25] for methanolic leaf extracts of Sardinian *P. lentiscus* (IC_50_ ≈ 40 µg/mL), despite the exclusive use of water as the extraction solvent in the present work. In contrast, the ethyl acetate fraction investigated by Elloumi et al. [23] exhibited a markedly lower tyrosinase inhibitory activity (IC_50_ = 123 µg/mL), indicating reduced efficacy of this solvent system for the extraction of tyrosinase-modulating compounds.

With regard to tyrosinase inhibition, it is common knowledge that the dopachrome-based assay could easily give false positive results, due to the ability of phenolics, contained in raw extracts, to react with dopaquinone, thus acting as quinone scavengers and preventing dopachrome formation [40]. To overcome this issue, which is of maximum importance in the case of the phenolic-rich *P. lentiscus* extract, an alternative catechol/aromatic amine assay was employed, allowing discrimination between quinone-scavenging effects and true enzymatic inhibition. Using this approach, the *P. lentiscus* extract was confirmed to act as a “true” tyrosinase inhibitor, effectively preventing the formation of the characteristic purplish-blue adduct between 4-methyl-1,2-benzoquinone (arising from the enzyme action on methylcatechol) and the aromatic diamine.

Besides, a ‘true’ catecholase, that from *Cynara*, void of any tyrosinase (i.e., monophenolase) activity, was tested to establish if the extract acted on a pure diphenolase activity. The experiments confirmed that the diphenolase activity was strongly inhibited by the extract. It is worth noting that the *Cynara* extract was rich in phenolics, behaving as competing substrates towards 4-methylcatechol. The addition of generous amounts of insoluble crosslinked polyvinylpyrrolidone powder was necessary to adsorb such interfering compounds and measure the catecholase activity.

With regard to elastase inhibition, the IC_50_ value obtained for the aqueous extract (17.4 µg/mL) is consistent with that reported by Elloumi et al. [23] for the ethyl acetate fraction (IC_50_ = 19 µg/mL), while Chiocchio et al. [25] described a slightly stronger inhibitory activity for methanolic extracts (IC_50_ ≈ 7.17 µg/mL).

A particularly relevant finding of this study is the marked anti-hyaluronidase activity of the extract, which, to the best of our knowledge, has not been previously reported for *P. lentiscus*. The inhibition observed (IC_50_ 4.3 µg/mL) was substantially stronger than that of the reference compound oleanolic acid (IC_50_ 97.0 µg/mL), suggesting that the complex mixture of phenolic constituents exerts a cooperative effect on enzyme modulation. Conversely, no collagenase inhibition was detected, indicating a degree of selectivity in the enzyme-targeting profile of the extract. This selective pattern may be attributed to the specific structural features of the dominant phenolic compounds and their differential affinity for enzyme active sites.

Several of the identified constituents, including myricetin, quercetin, galloyl quinic acids, and epigallocatechin gallate, have been individually reported to possess antioxidant activity and wound healing properties [45,46]. More detailed investigations have been conducted on specific flavonol glycosides such as quercetin-3-*O*-rhamnoside and myricetin-3-*O*-rhamnoside, which here accounted for approximately 10.2% and 2.0% of the total polyphenolic content, demonstrating their ability to inhibit tyrosinase and elastase [23,47]. In addition to their enzyme-inhibitory properties, quercetin-3-*O*-rhamnoside and myricetin-3-*O*-rhamnoside have also been reported to significantly promote wound healing in vivo, enhancing wound closure, re-epithelialization, collagen deposition, and attenuation of inflammatory markers, thereby supporting their role in skin regeneration processes [47].

Furthermore, the high abundance of galloylated compounds may substantially contribute to the enzyme inhibition mechanisms observed. Galloyl derivatives are known for their strong protein-binding properties and high affinity toward enzyme active sites, which have been associated with broad-spectrum inhibitory activity [42,43,44,48]. Consistently, *P. lentiscus* extracts rich in galloylated compounds have been reported to inhibit multiple enzymatic systems, including inflammatory mediators, carbohydrate- and lipid-digesting enzymes, as well as enzymes involved in microbial and viral processes [22,27,33,49,50].

These findings indicate that, while individual compounds contribute to the observed bioactivity, they do not fully account for the magnitude of the effects detected. Accordingly, the biological activities observed in the present study are unlikely to be attributable to a single constituent. Rather, they appear to reflect the synergistic action of the phytocomplex, in which multiple phenolic compounds interact through complementary and overlapping mechanisms, resulting in enhanced overall efficacy.

Taken together, these results suggest that aqueous residues obtained after *P. lentiscus* hydrodistillation retain a high functional value and may serve as a multifunctional ingredient for dermocosmetic applications. The combination of essential oil production with the recovery of a phenolic-rich aqueous extract offers a unique opportunity to fully exploit *P. lentiscus* biomass, maximizing resource efficiency while minimizing environmental impact. Such extracts could be integrated into cosmetic formulations either alone or in combination with the corresponding essential oils, potentially providing complementary activities through both volatile and non-volatile fractions. Overall, this study supports the use of *P. lentiscus* aqueous extracts as sustainable, effective, and safe candidates for anti-aging skin applications and encourages further formulation and in vivo investigations.

## 4. Materials and Methods

### 4.1. Reagents and Standards

All the chemicals used were of analytical grade. Methanol and 85% *w*/*w* phosphoric acid were purchased from Sigma-Aldrich (Steinheim, Germany). LC-MS grade acetonitrile, ethyl acetate, formic acid, and water were purchased from Merck (Darmstadt, Germany). Cross-linked polyvinylpyrrolidone, 4-amino-*N*,*N*-diethylaniline sulfate, and 4-methylcatechol were from Fluka (Buchs, Switzerland). Standards of gallic acid, gallocatechin, epigallocatechin gallate, myricetin 3-*O*-rutinoside, myricetin 3-*O*-rhamnoside, quercetin, quercetin-3-*O*-rhamnoside, and kaempferol were purchased from Extrasynthese (Genay Cedex, France) and TransMIT (Giessen, Germany). Ultrapure water (18 MΩ·cm) was obtained with a Milli-Q Advantage A10 System apparatus (Millipore, Milan, Italy).

### 4.2. Plant Material

Samples of *Pistacia lentiscus* L. were collected in March 2021 in the Sulcis region, south-western Sardinia (Monte Orbai, in the municipality of Villamassargia, with coordinates 39°14′09.7″ N 8°43′32.8″ E). Within the area, the aerial parts of three different plants were harvested. Following collection, the samples were cleaned to remove woody material, weighed, and air-dried for 20 days in the dark at a controlled temperature (15–20 °C). The dried plant material was then subjected to hydro-distillation in accordance with pharmacopeial procedures [51]. Briefly, the samples were finely chopped and subjected to hydrodistillation using a Clevenger-type apparatus for 3 h at 100 °C. Plant material was mixed with distilled water at a ratio of 1:10 (*w*/*v*), ensuring complete immersion. The brownish-green aqueous decoction obtained after distillation was filtered to remove residual plant material and stored in the frozen state (−80 °C) until further analysis.

### 4.3. High-Resolution HPLC-ESI-QTOF-MS-MS and HPLC-PDA Analysis

The qualitative investigation of the plant extracts was performed by an ion mobility Q-TOF LC/MS system according to De Luca et al. [52], using a 1290 Infinity II UPLC equipped with a 6560 IM-QTOF (Agilent Technologies Inc., Palo Alto, CA, USA). The electrospray ionization (ESI) source in negative ion mode was used to perform all the experiments, and the mass spectra were acquired by full range acquisition covering the *m*/*z* range of 40–1300. Chromatographic separation was performed on a Kinetex EVO C18 column (150 × 2.1 mm, 1.7 µm 100 Å, Phenomenex, Castel Maggiore, Italy) using a mobile phase consisting of 0.1% formic acid and acetonitrile + 0.1% formic acid mixed with an appropriate gradient elution at a flow rate of 0.3 mL/min. The injection volume was 4 µL. Data acquisition and processing were done using Agilent MassHunter Workstation Acquisition software v. B.09.00 (Agilent Technologies). ESI/QTOF MS data were then analyzed using the molecular feature extraction algorithm of the MassHunter Workstation Qualitative Analysis software v. 10.0 (Agilent Technologies). The MassHunter METLIN metabolite PCDLdatabase B.08.00 (Agilent Technologies) and Sirius^®^ software version 4.7.4 were used for the tentative identification of the metabolites and to predict fragmentation and molecular formulae [28,29].

The quantitative analysis of targeted phenolic compounds was carried out using an HPLC-PDA method as described by De Luca et al. [53] using an Agilent 1260 Infinity II HPLC system and an Agilent G4212B photodiode array detector. (Agilent Technologies). The separation was obtained with a Kinetex EVO C18 column (150 × 4.60 mm, 2.6 μm, Phenomenex) using 0.22 M phosphoric acid and acetonitrile as mobile phase properly mixed in gradient elution, at a constant flow rate of 0.8 mL/min. The injection volume was 10 μL. The chromatograms and spectra were elaborated with an OpenLab V. 2.51 data system (Agilent Technologies), and phenolic compounds were detected and quantified according to the main classes: flavonoids at 360 nm, and hydroxybenzoic acids at 280 nm. For the quantitative analysis, *P. lentiscus* water residues remaining after hydrodistillation were diluted 1:1 *v*/*v* with 0.22 M H_3_PO_4_, filtered with a 0.22 μm CA syringe and injected into the LC-PDA system. Phenolic compounds content was expressed as mg/L.

### 4.4. Tyrosinase Assay

The inhibition of tyrosinase activity by *P. lentiscus* WEDR was determined by using 3,4-dihydroxyphenylalanine (L-DOPA) as substrate. The reaction mixture contained 50 mM phosphate buffer (pH 6.8), mushroom tyrosinase (Sigma Chemical Co., Milan, Italy), with or without a plant extract solution. Then, L-DOPA (0.5 mM) was added to the mixture, and the activity was determined by following the increase in absorbance at 492 nm resulting from the formation of the dopachrome product. Kojic acid was used as a positive control.

To validate the obtained results, the method of Valgimigli et al. [54] was also used with minor modifications. The reaction mixture contained, in a final volume of 2 mL, 400 µL mushroom tyrosinase (Sigma Chemical Co., Milan, Italy), 10 mM 4-methylcatechol, and 5 mM 4-amino-*N*,*N*-diethylaniline sulfate in 50 mM potassium citrate buffer, pH 6. A mother solution of the amine (50 mM) had been previously prepared in 0.1 M citric acid to avoid autoxidation. The solution was gently stirred for 10 min, then 2 mL of ethyl acetate was added, and the mixture was vortexed for 10 min. The organic upper phase was drawn with a Pasteur pipette, dried with anhydrous sodium sulfate and read in a glass semimicro cuvette at 600 nm.

To study the inhibiting activity of the *P. lentiscus* WEDR, established volumes of buffer in the assay solution were substituted with identical volumes of the extract. To exclude the presence of contaminating laccase in the commercial tyrosinase preparation used, other experiments were performed as depicted above, where no methylcatechol was present in the assay solution, but only the enzyme and aminodiethylaniline (which is not a tyrosinase substrate but is oxidized by laccase to form a purplish-red cationic semiquinone).

In another series of experiments, mushroom tyrosinase was substituted with a raw catecholase preparation from artichoke (*Cynara scolymus*, cultivar ‘Spinoso Sardo’). All the experimental details were identical to those described for mushroom tyrosinase, except for the use of excess cross-linked polyvinylpyrrolidone powder during artichoke hearts homogenate preparation, and subsequent filtration by filter paper prior to use.

### 4.5. Elastase Assay

Elastase inhibition was assayed by monitoring the release of p-nitroaniline during cleavage of the substrate N-succ-(Ala)3-nitroanilide (SANA) by the action of the enzyme, using the method described by Chompoo et al. [55], with slight modifications. The assay was performed in 0.1 M Tris-HCl buffer (pH 8.0). Porcine pancreatic elastase (3.3 µg/mL) was incubated with or without the extract for 20 min, and after incubation, the substrate (1.6 mM) was added, and the enzyme activity was monitored at 410 nm. Oleanolic acid was used as a positive control.

### 4.6. Collagenase Assay

Collagenase from *Clostridium histolyticum* was dissolved in Tricine buffer (0.05 M, pH 7.5) supplemented with NaCl (0.04 M) and CaCl_2_ (0.01 M). The enzyme solution (1 U/mL) was incubated for 15 min with or without the extract. The reaction was then initiated by adding the synthetic substrate N-(3-[2-Furyl]-acryloyl)-Leu-Gly-Pro-Ala (FALGPA) to reach the final concentration of 0.8 mM, and absorbance at 340 nm was recorded. Epigallocatechin was used as a positive control.

### 4.7. Hyaluronidase Assay

Hyaluronidase activity was assessed according to the procedure described by Chompoo et al. [55]. A 5 µL aliquot of extract solution was mixed with 100 µL of the enzyme preparation (1.5 U), consisting of 20 mM sodium phosphate buffer (pH 7.0) supplemented with 77 mM NaCl and 0.01% bovine serum albumin (BSA). The mixture was incubated at 37 °C for 10 min. Subsequently, 100 µL of the substrate solution composed of 0.03% hyaluronic acid in 300 mM sodium phosphate buffer (pH 5.35) was added. The reaction was continued for an additional 45 min at 37 °C. Undigested hyaluronic acid was then precipitated using 1 mL of acid albumin solution (0.1% BSA in 24 mM sodium acetate and 79 mM acetic acid, pH 3.75). After incubation for 10 min at room temperature, absorbance was measured at 600 nm. Oleanolic acid was used as a positive reference compound.

### 4.8. ABTS Assay

The free radical 2,2′-Azinobis-(3-Ethylbenzothiazoline-6-Sulfonic Acid (ABTS^•+^) was produced by reacting 7 mM ABTS with 2.45 mM potassium persulfate in aqueous solution and keeping it in the dark at room temperature for 24 h before use. Subsequently, an aliquot of this mixture was diluted to obtain an absorbance of approximately 0.700 ± 0.05. Samples of each extract (10 μL) were added to 1 mL of ABTS·+, and the absorbance at 734 nm was recorded after 1 min incubation. For this assay, 6-hydroxy-2,5,7,8-tetramethylchromane-2-carboxylic acid (Trolox) was used as a standard reference.

### 4.9. Total Polyphenol Content

Total polyphenol content was determined by the Folin–Ciocalteu reagent. Briefly, 10 μL of the extract was dissolved in 50 μL of the Folin–Ciocalteu reagent and 790 μL of distilled water. The solution was mixed and incubated at room temperature for 1 min. Subsequently, 150 μL of 20% sodium carbonate solution was added. The solution was shaken and then incubated at room temperature in the dark for 45 min. The absorbance of all samples was measured at 750 nm. A calibration curve was plotted using gallic acid as a standard. The results were expressed as mg of gallic acid equivalents (GAE) per g of dry weight (dw).

### 4.10. Total Flavonoid Content

The flavonoid content in the extract was determined by the aluminium nitrate colorimetric method. An aliquot of 10 μL of sample solution was mixed with 20 μL of 10% (*w*/*v*) aluminium nitrate, 20 μL of 1 M sodium acetate and 850 μL of 80% ethanol. After incubation at room temperature for 40 min, the absorbance of the reaction mixture was measured at 415 nm. Different concentrations of quercetin solution were used for calibrations, and results were expressed as mg of quercetin equivalents (QE) per g of dw.

### 4.11. Cell Viability Assay

The cellular cytotoxicity of the extract was investigated using a 3-(4,5-dimethylthiazol-2-yl)-2,5-diphenyl-tetrazolium bromide (MTT) assay. The HaCaT cell line of human keratinocytes was obtained from CLS-Cell Line Services in Eppelheim, Germany. The cells were exposed for 24 h to extract at concentrations ranging from 30 to 500 µg/mL. Then, MTT reagent (0.5 mg/mL in DMEM) was added to each well. The plate was incubated for 3 h at 37 °C. Then, 100 μL of DMSO solvent was added, and the absorbance was determined at 570 nm using a microplate reader (VANTAstar_BMG LABTECH GmbH, Ortenberg, Germany).

### 4.12. Intracellular ROS Levels

The cellular ROS levels were determined using the DCFH-DA method described by Era et al. [39] with slight modifications. HaCaT cells were treated with various concentrations of extract (0–200 µg/mL) for 24 h. Then, the cells were incubated with DCFH-DA (10 µM) at 37 °C for 30 min. After incubation, 2 mM H_2_O_2_ was added to the wells, and the fluorescence intensity of DCF was measured immediately using a fluorescent plate reader with an excitation wavelength of 485 nm and an emission wavelength of 530 nm, taking readings at 5 min intervals for 120 min.

### 4.13. Statistical Analysis

Data are expressed as mean ± standard deviation (SD). One-way ANOVA and Tukey’s post hoc test were performed for group comparison using GraphPad Prism software v. 8 (San Diego, CA, USA). A *p*-value of less than 0.05 was considered statistically significant.

## 5. Conclusions

In conclusion, the present study demonstrates that aqueous residues obtained from *Pistacia lentiscus* leaf hydrodistillation constitute a phenolic-rich and biologically active by-product with multitarget anti-aging potential. The combined antioxidant capacity, enzyme-inhibitory activity, and favorable safety profile support the valorization of this sustainable extract as a multifunctional ingredient capable of acting at different skin levels, from the modulation of epidermal tyrosinase activity to the inhibition of elastase and hyaluronidase in deeper dermal compartments, for skin-related and dermocosmetic applications.

## Figures and Tables

**Table 3:** Total phenolic and flavonoid contents and antioxidant activity of *P. lentiscus* WEDR.

Sample	Total Phenolic mg GAE/g dw	Flavonoid mg QE/g dw	ABTS Scavenging IC_50_ Values (μg/mL)
WEDR	212.8 ± 9.9	32.7 ± 4.4	6.33 ± 0.02
Trolox °			3.4 ± 0.3

° [38].

**Table 4:** Inhibition of *P. lentiscus* WEDR expressed as IC_50_ values (µg/mL). Standard compounds are kojic acid for tyrosinase and oleanolic acid for elastase and hyaluronidase.

Sample	IC_50_ (µg/mL)		
	Tyrosinase	Elastase	Hyaluronidase
WEDR	33.8 ± 0.3	17.4 ± 1.1	4.3 ± 0.3
Kojic acid *	17.9 ± 0.98	-	-
Oleanolic acid	-	11.8 ± 0.6	97.0 ± 4.2

* [39].

## Data Availability

The original contributions presented in this study are included in the article/Supplementary Materials. Further inquiries can be directed to the corresponding author.

## Supplementary Materials

The following supporting information can be downloaded at https://www.mdpi.com/article/10.3390/plants15071013/s1. Table S1: raw fluorescence data for ROS levels in HaCat cells reported in Figure 3.

## References

[1] Liang Y., Su W., Wang F. (2023). Skin Ageing: A Progressive, Multi-Factorial Condition Demanding an Integrated, Multilayer-Targeted Remedy. Clin. Cosmet. Investig. Dermatol..

[2] Shin J.W., Kwon S.H., Choi J.Y., Na J.I., Huh C.H., Choi H.R., Park K.C. (2019). Molecular Mechanisms of Dermal Aging and Antiaging Approaches. Int. J. Mol. Sci..

[3] Pintus F., Floris S., Fais A., Era B., Kumar A., Gatto G., Uriarte E., Matos M.J. (2022). Hydroxy-3-Phenylcoumarins as Multitarget Compounds for Skin Aging Diseases: Synthesis, Molecular Docking and Tyrosinase, Elastase, Collagenase and Hyaluronidase Inhibition, and Sun Protection Factor. Molecules.

[4] Zhang M., Lin Y., Han Z., Huang X., Zhou S., Wang S., Zhou Y., Han X., Chen H. (2024). Exploring Mechanisms of Skin Aging: Insights for Clinical Treatment. Front. Immunol..

[5] Pisano L., Turco M., Supuran C.T. (2024). Biomedical Applications of Tyrosinases and Tyrosinase Inhibitors. Enzymes.

[6] Manjia J.N., Njoya E.M., Harishchander A., Munvera A.M., Ogundolie F.A., Mkounga P., Mcgaw L.J., Njayou F.N., Moundipa P.F. (2024). Anti-Elastase, Anti-Tyrosinase, and Anti-Inflammatory Activities of Three Compounds Isolated from *Psorospermum aurantiacum*: In Silico and In Vitro Assays. Rev. Bras. Farmacogn..

[7] Wu S., Zhou X., Jin Z., Cheng H. (2023). Collagenases and Their Inhibitors: A Review. Collagen Leather.

[8] Lu J., Zhao Z., Pan L., Wu H., Wang S., Tong X., Wu S. (2025). Hyaluronidase: Structure, Mechanism of Action, Diseases and Therapeutic Targets. Mol. Biomed..

[9] Jesus A., Ratanji S., Cidade H., Sousa E., Cruz M.T., Oliveira R., Almeida I.F. (2025). Phenolics as Active Ingredients in Skincare Products: A Myth or Reality?. Molecules.

[10] Wang Y., Hao M.M., Sun Y., Wang L.F., Wang H., Zhang Y.J., Li H.Y., Zhuang P.W., Yang Z. (2018). Synergistic Promotion on Tyrosinase Inhibition by Antioxidants. Molecules.

[11] He X., Gao X., Guo Y., Xie W. (2024). Research Progress on Bioactive Factors against Skin Aging. Int. J. Mol. Sci..

[12] IRMNG (2021). Anacardiaceae R. Brown, 1818. https://www.irmng.org/aphia.php?p=taxdetails&id=114601.

[13] Oliveira M., Hoste H., Custódio L. (2021). A Systematic Review on the Ethnoveterinary Uses of Mediterranean Salt-Tolerant Plants: Exploring Its Potential Use as Fodder, Nutraceuticals or Phytotherapeutics in Ruminant Production. J. Ethnopharmacol..

[14] Salhi N., Bouyahya A., Fettach S., Zellou A., Cherrah Y. (2019). Ethnopharmacological Study of Medicinal Plants Used in the Treatment of Skin Burns in Occidental Morocco (Area of Rabat). S. Afr. J. Bot..

[15] Bouasla A., Bouasla I. (2017). Ethnobotanical Survey of Medicinal Plants in Northeastern of Algeria. Phytomedicine.

[16] Bullitta S., Piluzza G., Viegi L. (2007). Plant Resources Used for Traditional Ethnoveterinary Phytotherapy in Sardinia (Italy). Genet. Resour. Crop Evol..

[17] Bouakline H., Elkabous M., Ziani I., Karzazi Y., Tahani A., El Bachiri A. (2023). Antioxidative Activity of *Pistacia lentiscus* Leaf Extract Main Components: Experimental and Theoretical Study. Mater. Today Proc..

[18] Balan K.V., Prince J., Han Z., Dimas K., Cladaras M., Wyche J.H., Sitaras N.M., Pantazis P. (2007). Antiproliferative Activity and Induction of Apoptosis in Human Colon Cancer Cells Treated in Vitro with Constituents of a Product Derived from *Pistacia lentiscus* L. var. *chia*. Phytomedicine.

[19] Spyridopoulou K., Tiptiri-Kourpeti A., Lampri E., Fitsiou E., Vasileiadis S., Vamvakias M., Bardouki H., Goussia A., Malamou-Mitsi V., Panayiotidis M.I. (2017). Dietary Mastic Oil Extracted from *Pistacia lentiscus* var. *chia* Suppresses Tumor Growth in Experimental Colon Cancer Models. Sci. Rep..

[20] Magkouta S., Stathopoulos G.T., Psallidas I., Papapetropoulos A., Kolisis F.N., Roussos C., Loutrari H. (2009). Protective Effects of Mastic Oil from *Pistacia lentiscus* Variation *chia* against Experimental Growth of Lewis Lung Carcinoma. Nutr. Cancer.

[21] Janakat S., Al-Merie H. (2002). Evaluation of Hepatoprotective Effect of *Pistacia lentiscus*, *Phillyrea latifolia* and *Nicotiana glauca*. J. Ethnopharmacol..

[22] Bouakline H., Bouknana S., Merzouki M., Ziani I., Challioui A., Bnouham M., Tahani A., El Bachiri A. (2024). The Phenolic Content of *Pistacia lentiscus* Leaf Extract and Its Antioxidant and Antidiabetic Properties. Sci. World J..

[23] Elloumi W., Maalej A., Ortiz S., Michel S., Chamkha M., Boutefnouchet S., Sayadi S. (2022). *Pistacia lentiscus* L. Distilled Leaves as a Potential Cosmeceutical Ingredient: Phytochemical Characterization, Transdermal Diffusion, and Anti-Elastase and Anti-Tyrosinase Activities. Molecules.

[24] Labhar A., El-Mernissi Y., Alla K.A., Jahjah S., Benamari O., Zouhri A., Labhar F., Siddique F., Jamali C.A., Abdellah E. (2025). Influence of the Seasons on the Chemical Composition and Biological Properties of *Pistacia lentiscus* L. Essential Oil in the Mediterranean Region. Sci. Rep..

[25] Chiocchio I., Mandrone M., Sanna C., Maxia A., Tacchini M., Poli F. (2018). Screening of a Hundred Plant Extracts as Tyrosinase and Elastase Inhibitors, Two Enzymatic Targets of Cosmetic Interest. Ind. Crops Prod..

[26] Bampouli A., Kyriakopoulou K., Papaefstathiou G., Louli V., Krokida M., Magoulas K. (2014). Comparison of Different Extraction Methods of *Pistacia lentiscus* var. *chia* Leaves: Yield, Antioxidant Activity and Essential Oil Chemical Composition. J. Appl. Res. Med. Aromat. Plants.

[27] El Allaoui H., Haboubi K., El Ahmadi K., Bouhrim M., ElAbdouni A., Eto B., Shahat A.A., Herqash R.N., El Bestrioui M., Zouaoui Z. (2025). Comprehensive Assessment of Antioxidant, Antidiabetic, and Anti-Glycation Properties of Aqueous and Methanolic Extracts from *Pistacia lentiscus* L. Leaves: A Potential Natural Source for Managing Oxidative Stress and Diabetes-Related Complications. Front. Pharmacol..

[28] Dührkop K., Fleischauer M., Ludwig M., Aksenov A.A., Melnik A.V., Meusel M., Dorrestein P.C., Rousu J., Böcker S. (2019). SIRIUS 4: A Rapid Tool for Turning Tandem Mass Spectra into Metabolite Structure Information. Nat. Methods.

[29] Hoffmann M.A., Nothias L.F., Ludwig M., Fleischauer M., Gentry E.C., Witting M., Dorrestein P.C., Dührkop K., Böcker S. (2021). High-Confidence Structural Annotation of Metabolites Absent from Spectral Libraries. Nat. Biotechnol..

[30] KNApSAcK Core System. https://www.knapsackfamily.com/KNApSAcK/.

[31] Blaženović I., Kind T., Ji J., Fiehn O. (2018). Software Tools and Approaches for Compound Identification of LC-MS/MS Data in Metabolomics. Metabolites.

[32] Pacifico S., Piccolella S., Marciano S., Galasso S., Nocera P., Piscopo V., Fiorentino A., Monaco P. (2014). LC-MS/MS Profiling of a Mastic Leaf Phenol Enriched Extract and Its Effects on H_2_O_2_ and Aβ(25-35) Oxidative Injury in SK-B-NE(C)-2 Cells. J. Agric. Food Chem..

[33] Boucheffa S., Sobhi W., Attoui A., Selli S., Kelebek H., Semmeq A., Benguerba Y. (2022). Effect of the Main Constituents of *Pistacia lentiscus* Leaves against the DPPH Radical and Xanthine Oxidase: Experimental and Theoretical Study. J. Biomol. Struct. Dyn..

[34] Liu X., Fu Y., Ma Q., Yi J., Cai S. (2021). Anti-Diabetic Effects of Different Phenolic-Rich Fractions from *Rhus chinensis* Mill. Fruits in Vitro. eFood.

[35] Negri G., Tabach R. (2013). Saponins, Tannins and Flavonols Found in Hydroethanolic Extract from *Periandra dulcis* Roots. Rev. Bras. Farmacogn..

[36] El Bishbishy M.H., Gad H.A., Aborehab N.M. (2020). Chemometric Discrimination of Three *Pistacia* Species via Their Metabolic Profiling and Their Possible in Vitro Effects on Memory Functions. J. Pharm. Biomed. Anal..

[37] Gu D., Yang Y., Bakri M., Chen Q., Xin X., Aisa H.A. (2013). A LC/QTOF-MS/MS Application to Investigate Chemical Compositions in a Fraction with Protein Tyrosine Phosphatase 1B Inhibitory Activity from *Rosa rugosa* Flowers. Phytochem. Anal..

[38] Di Petrillo A., González-Paramás A.M., Era B., Medda R., Pintus F., Santos-Buelga C., Fais A. (2016). Tyrosinase Inhibition and Antioxidant Properties of *Asphodelus microcarpus* Extracts. BMC Complement. Altern. Med..

[39] Era B., Floris S., Sogos V., Porcedda C., Piras A., Medda R., Fais A., Pintus F. (2021). Anti-Aging Potential of Extracts from *Washingtonia filifera* Seeds. Plants.

[40] Rescigno A., Sollai F., Pisu B., Rinaldi A., Sanjust E. (2002). Tyrosinase Inhibition: General and Applied Aspects. J. Enzym. Inhib. Med. Chem..

[41] Sehaki C., Jullian N., Ayati F., Fernane F., Gontier E. (2023). A Review of *Pistacia lentiscus* Polyphenols: Chemical Diversity and Pharmacological Activities. Plants.

[42] Owegie O.C., Kennedy Q.P., Davizon-Castillo P., Yang M. (2025). Thiol Isomerases: Enzymatic Mechanisms, Models of Oxidation, and Antagonism by Galloylated Polyphenols. Antioxidants.

[43] Cao Y., Xiong Y.L. (2017). Binding of Gallic Acid and Epigallocatechin Gallate to Heat-Unfolded Whey Proteins at Neutral PH Alters Radical Scavenging Activity of in Vitro Protein Digests. J. Agric. Food Chem..

[44] Mutlu D., Doldur A.N., Cavdar D., Ozkan G., Ceylan F.D. (2025). Current Methodologies for Assessing Protein–Phenolic Interactions. Discov. Food.

[45] Moghadam S.E., Ebrahimi S.N., Salehi P., Farimani M.M., Hamburger M., Jabbarzadeh E. (2017). Wound Healing Potential of Chlorogenic Acid and Myricetin-3-O-β-Rhamnoside Isolated from *Parrotia persica*. Molecules.

[46] Süntar I.P., Akkol E.K., Yalçin F.N., Koca U., Keleş H., Yesilada E. (2010). Wound Healing Potential of *Sambucus ebulus* L. Leaves and Isolation of an Active Component, Quercetin 3-O-Glucoside. J. Ethnopharmacol..

[47] Elloumi W., Mahmoudi A., Ortiz S., Boutefnouchet S., Chamkha M., Sayadi S. (2022). Wound Healing Potential of Quercetin-3-O-Rhamnoside and Myricetin-3-O-Rhamnoside Isolated from *Pistacia lentiscus* Distilled Leaves in Rats Model. Biomed. Pharmacother..

[48] Wang X., Zhang J., Lei F., Liang C., Yuan F., Gao Y. (2014). Covalent Complexation and Functional Evaluation of (−)-Epigallocatechin Gallate and α-Lactalbumin. Food Chem..

[49] Boutemine I.M., Amri M., Dorgham K., Amir Z.C., Benazzouz S., Ameur F., Layaida K., Yssel H., Touil-Boukoffa C. (2021). Beneficial Role of *Pistacia lentiscus* Aqueous Extract in Experimental Colitis: Anti-Inflammatory and Potential Therapeutic Effects. Inflammopharmacology.

[50] Samandar F., Amiri Tehranizadeh Z., Saberi M.R., Chamani J. (2022). 1,2,3,4,6-Pentagalloyl Glucose of *Pistacia lentiscus* Can Inhibit the Replication and Transcription Processes and Viral Pathogenesis of SARS-CoV-2. Mol. Cell. Probes.

[51] Council of Europe (2010). European Pharmacopoeia.

[52] De Luca M., Tuberoso C.I.G., Pons R., García M.T., Morán M.d.C., Ferino G., Vassallo A., Martelli G., Caddeo C. (2023). Phenolic Fingerprint, Bioactivity and Nanoformulation of *Prunus spinosa* L. Fruit Extract for Skin Delivery. Pharmaceutics.

[53] De Luca M., Lucchesi D., Tuberoso C.I.G., Fernàndez-Busquets X., Vassallo A., Martelli G., Fadda A.M., Pucci L., Caddeo C. (2022). Liposomal Formulations to Improve Antioxidant Power of Myrtle Berry Extract for Potential Skin Application. Pharmaceutics.

[54] Valgimigli L., Sanjust E., Curreli N., Rinaldi A., Pedulli G.F., Rescigno A. (2001). Photometric Assay for Polyphenol Oxidase Activity in Olives, Olive Pastes, and Virgin Olive Oils. J. Am. Oil Chem. Soc..

[55] Chompoo J., Upadhyay A., Fukuta M., Tawata S. (2012). Effect of Alpinia Zerumbet Components on Antioxidant and Skin Diseases-Related Enzymes. BMC Complement. Altern. Med..

