# Supplementary material for: Valorization of Pistacia lentiscus L. Hydrodistillation By-Products: Phytochemical Profile and Multitarget Anti-Aging Activity of an Aqueous Extract
Source: Plants (Basel). 2026 Mar 26;15(7):1013. doi: 10.3390/plants15071013 (PMC13074500; doi:10.3390/plants15071013)
Supplement: Supplementary file 1 [file plants-15-01013-s001.zip › plants-4193559-supplementary.pdf]

TABLE OF CONTENTS

1. Biology

- Table 1S. Raw fluorescence data for ROS levels in HaCat cells reported in Figure 3

NT	T	30 μg/mL	100 μg/mL	150 μg/mL	200 μg/mL
29398	108547	104011	89984	78911	89233
36593	114654	108754	82489	95378	81540
35764	123750	108178	101949	82595	87553
34687	109135	110274	83124	87391	77669
37008	121875	101182	84980	96378	81731
41670	123324	105645	99438	89920	86038
